# CD4 T Cells Mediate Both Positive and Negative Regulation of the Immune Response to HIV Infection: Complex Role of T Follicular Helper Cells and Regulatory T Cells in Pathogenesis

**DOI:** 10.3389/fimmu.2014.00681

**Published:** 2015-01-06

**Authors:** Chansavath Phetsouphanh, Yin Xu, John Zaunders

**Affiliations:** ^1^Centre for Applied Medical Research, Kirby Institute, St Vincent’s Hospital, University of New South Wales, Sydney, NSW, Australia

**Keywords:** CD4, regulatory T cells, T follicular helper cells, HIV infections, lymphoid tissue

## Abstract

HIV-1 infection results in chronic activation of cells in lymphoid tissue, including T cells, B-cells, and myeloid lineage cells. The resulting characteristic hyperplasia is an amalgam of proliferating host immune cells in the adaptive response, increased concentrations of innate response mediators due to viral and bacterial products, and homeostatic responses to inflammation. While it is generally thought that CD4 T cells are greatly depleted, in fact, two types of CD4 T cells appear to be increased, namely, regulatory T cells (Tregs) and T follicular helper cells (Tfh). These cells have opposing roles, but may both be important in the pathogenic process. Whether Tregs are failing in their role to limit lymphocyte activation is unclear, but there is no doubt now that Tfh are associated with B-cell hyperplasia and increased germinal center activity. Antiretroviral therapy may reduce the lymphocyte activation, but not completely, and therefore, there is a need for interventions that selectively enhance normal CD4 function without exacerbating Tfh, B-cell, or Treg dysfunction.

## Introduction

The pathogenesis of CD4 T cell decline during chronic HIV-1 infection is slow and complex. It typically begins with a decrease of CD4 cell counts in peripheral blood, from a median of approximately 800 cells/μl to a median of 500 cells/μl during the first few weeks of primary infection (1–3), but is then followed by a much slower rate of decline over several years (1), eventually leading to opportunistic infections. CD4 cell counts in blood may not accurately reflect cell numbers in secondary lymphoid tissue, since treatment commenced during primary infection leads to a very rapid increase of CD4 cell counts that cannot be accounted for by new production of CD4 T cells and is most likely due to redistribution of resting cells that had been sequestered in lymph nodes (4, 5), as had been suggested for treatment commenced during chronic infection (6–8).

Therefore, depletion within lymphoid tissue early in infection is not so clear. Contradictory results from the SIV model of infection in rhesus macaques suggest either very high levels of infection and loss of CD4 T cells, particularly from gut associated lymphoid tissue (GALT) during primary infection (9, 10), as against sequestration and even proliferation of CD4 T cells in secondary lymphoid tissue during early chronic infection (11–13). In fact, increased rates of proliferation of both CD4 and CD8 T cells are a hallmark of chronic HIV-1 infection (14–16). This increased proliferation begins at the earliest stages of primary HIV-1 infection (5) and is associated with a CD4 response to viral antigens (17). An analogous proliferative response of CD4 and CD8 T cells to vaccinia virus was also clearly observed around day 13 post-inoculation in healthy adults (18). However, in the response to vaccinia virus, as neutralizing antibodies titers increased by day 21 post-inoculation, activation, and proliferation of CD4 and CD8 T cells were rapidly terminated (18), and this was later confirmed using tetramers to identify antigen-specific CD8 T cells (19).

Taken together, these results suggest that changes in CD4 cell numbers during HIV-1 infection are a complex summation of proliferating, but mostly short-lived, CD4 T cells, loss of virally infected cells, changes in trafficking, and feedback regulation to limit responses. These processes will occur in secondary lymphoid tissue and GALT, since they are the major sites of viral replication and antigen deposition (20, 21) and hence antigen presentation. In particular, germinal center (GC) hyperplasia and hypergammaglobulinemia are also absolutely characteristic of established HIV-1 infection (22). For most of the time that HIV-1 infection has been studied, it has been believed that very few antigen-specific CD4 T cells can be found in patients, presumably due to preferential targeting of these cells by virus (23), except that they are somehow protected in rare long-term non-progressors (LTNP) and even rarer elite controllers (EC) [reviewed in Ref. (24)]. Yet, this view of a paucity of HIV-specific CD4 T cells did not take into account the extremely high titers of HIV-specific IgG antibodies found in virtually all patients (22), beginning early in primary infection (25). These antibodies strongly suggest vigorous CD4 T cell help for B-cell responses, consistent with the greatly increased numbers of GCs associated with HIV-1 infection.

This review will discuss the regulatory environment within HIV-1 infected lymphoid tissue, with particular reference to the role of T follicular helper cells (Tfh) in driving B-cell activation and the role of regulatory T cells (Tregs) in countering lymphocyte activation. Since T cell and B-cell activation and proliferation appear to be unrelenting during early chronic infection, the evidence suggests that the positive regulation by Tfh prevails, and that Treg suppression is insufficient to prevent this.

## Anti-Viral CD4 T Cell Responses

CD4 T cell responses are pivotal in the development of effective cellular and humoral immunity against viral infections (26). The crucial role of CD4 T cells was firmly documented in murine models, where adoptive transfer of lymphocytic choriomeningitis virus (LCMV) specific CD4 T cells into mice with chronic infection restored function to exhausted CD8+ T cells and reduced viral burden (27, 28). Similarly in human cytomegalovirus (CMV) infection, loss of CD4 T cell function correlated with end-organ disease, and adoptive transfer of CMV-specific CD4 T cells into infected patients leads to reduction in viremia and immune restoration (29, 30). In the case of HIV-1 infection, LTNP and EC may control viral replication with the help of cytotoxic CD4 T lymphocytes specific for p24 (31, 32), and characteristically have CD4 T cells that vigorously proliferate in response to HIV-1 antigens, compared to non-proliferative CD4 T cells from subjects with progressive disease (24).

While exhaustion and dysfunction of anti-viral effector T cells have been suggested as a major factor in chronic viral infections, particularly the LCMV model in mice (33), the role of neutralizing antibodies, generated through CD4 help for B-cells in GCs may well be the ultimate determinant of outcome (34, 35). Recently, it has been reported in the LCMV model that late development of a neutralizing antibody response correlates with eventual clearance of the chronic infection, rather than T cell immunity (36). This clearance is associated with a slow development of viral antigen-specific Tfh (37). At the same time, a negative role for Tregs in anti-microbial responses in animal models, and outcome of these infections, is clearly established (38), and are highly likely to provide negative feedback regulation to limit tissue damage. Therefore, there are diverse effector and regulatory roles of CD4 T cells in the anti-viral response.

Human immunodeficiency virus (HIV) infection is a prime example of the clinical relevance of CD4 T cell loss, where progressive depletion of the T helper (Th) population leads to increased morbidity and eventual mortality if untreated. Depletion of CD4 T cells is believed to be mostly due to direct infection of this subset (21). However, loss of cells may also be due to chronic immune activation, secondary to chronic exposure to microbial products translocated across epithelial barriers depleted of CD4 T cells during primary HIV-1 infection (39) and alteration of homeostasis due to eventual fibrotic changes to lymphoid tissue (40). Direct anti-viral effector functions of human CD4 T cells are quite clear, particularly cytotoxic activity in HIV-1 and CMV infections, respectively (31, 32, 41, 42). However, the demonstrated cardinal role of the various subsets of CD4 T cells in experimental models of immune responses is to ensure optimal help to other lymphocytes, especially B-cells (Tfh subset of CD4 T cells) and CD8 T cells, as well as to recruit monocytes (Th1), eosinophils and basophils (Th2), and neutrophils (Th17), and also to limit responses (Tregs) (43).

## Treg Phenotypes and Mechanisms of Action

Regulatory T cell-mediated suppression of inflammation serves as a crucial mechanism in the prevention of autoimmune disorders and the control of negative regulation of inflammatory diseases. Tregs are indispensible for the maintenance of homeostasis of the immune system that limits the magnitude of effector responses and allows the establishment of immunological tolerance. Two main types of Tregs have been identified, they include natural (or thymic) and induced (or peripheral) Tregs and both play important roles in turning down immune responses (44, 45).

Naturally arising CD4 regulatory T cells (nTregs) develop in the thymus and are primarily engaged in dominant control of self-reactive T cells (46). The initial evidence in support of thymic generation of cells that can mediate immune tolerance through suppression of other cells materialized from studies of neonatal thymectomy (47), but differentiation of inducible Treg cells occurs in the periphery, mainly within lymphoid tissue including GALT (48), where peripheral Tregs have increased affinity to non-self Ags, e.g., allergens, food, and commensal micro-biota. IL-10 producing regulatory T cells, termed Tr1 cells, are another subset of CD4 T cells, which produce the anti-inflammatory cytokines IL-10 (49) and transforming growth factor-β (TGF-β), and are involved in down regulating immune responses toward allergens and various antigens such as nickel and insect venom, as well as controlling autoimmunity, and preventing allograft rejection and graft versus host disease (GvHD) (50).

The transcription factor Foxp3 has been identified as the master regulator of Treg differentiation (45). In humans, CD25 alone cannot distinguish Tregs from activated CD4 T cells, and staining for Foxp3 involved fixation and permeabilization, thus it was necessary to find an additional marker for the identification of Tregs. It was discovered in 2006 by Seddiki et al. that the IL7Rα (CD127) is expressed at low to intermediate levels on the surface of Tregs and the combination of CD25+ CD127lo can be used to distinguish Treg from other CD4 subsets; CD25+ CD127low Tregs contain high amounts of Foxp3 and can suppress immune responses *in vitro* (51, 52).

## Tregs in HIV Infection

Regulatory T cells have been associated with several roles in HIV infection, which may occur at different times during the infection process and may be affected by ongoing therapy. The negative roles of Tregs in HIV infection include inhibitory effects on effector T cells during early infection (53); may serve as possible targets for HIV replication (54); and may have the ability to suppress HIV-specific responses that can lead to inhibition of T cell responses to HIV and increase viral persistence, leading to immune exhaustion (55, 56). Possible beneficial roles of Tregs may be their ability to reduce immune activation (57–59), particularly in situations of increased lipopolysaccharide (LPS) concentrations (60), and this restriction of activation of CD4 T cells could limit their loss.

A subset of Tregs can express CCR5, at a level comparable to other conventional CD4 T cells (Zaunders et al. unpublished data), which makes them susceptible to HIV infection (61–63). Naïve Tregs (nTregs) are able to upregulate CCR5 and CXCR4 following TCR stimulation, and when compared to conventional effector T helper cells, Tregs are less susceptible to HIV R5 strain but more susceptible to X4 strain *in vitro* (61). However, it is doubtful whether Tregs are major targets of HIV *in vivo* due to the small absolute number of CCR5+ Tregs [approximately 20 cells/μl in peripheral blood; (Zaunders et al. unpublished data)], and the relatively small amount of HIV DNA found in Tregs from HIV+ subjects reflects this (63). Rather the majority of Tregs may serve a role in inhibiting viral replication in other target CD4 T cells during early infection, which may assist in preventing the initial spread of the virus from the mucosal sites to lymph nodes (64, 65).

Despite evidence of some Tregs being infected, their suppressive function is largely retained in chronic progressive HIV-infection, originally shown through depletion experiments (53, 55, 57, 66), but more recently through analysis of the function of purified Tregs (67, 68). However, in one study of a small number of HIV+ subjects with immune reconstitution disease following antiretroviral therapy (ART), Tregs exhibited reduced suppression, and at the same time, responder cells from the same patients were less able to be suppressed by Tregs from healthy controls, suggesting overall impairment of Treg suppression (69).

During chronic HIV infection, the absolute Treg numbers in peripheral blood declined, but the proportion of Tregs among CD4 T cells is increased, regardless of the phenotype that was used (54, 70). This suggests that there is relative resistance of Tregs to the cell-depleting effects of HIV, compared to other CD4 T cell subsets. In one study, there was a relatively low proportion of Tregs in HIV+ EC that correlated with slightly higher T cell activation (71), but in an earlier study, no such difference had been found (18, 72). Other studies have shown that absolute numbers of Tregs in LTNP was similar to progressors, but frequencies were much lower than uninfected controls (62, 67, 73).

Accumulation of Tregs relative to conventional CD4 T cells during HIV infection could be explained by several mechanisms, which may include an increase in the proportion of CD25+ FoxP3+ cells regressing the thymus in HIV-infected individuals (74–76). Second, preferential survival and proliferation of Tregs may result from decreased sensitivity to TCR re-stimulation compared to non-Tregs, and a substantial resistance to activation-induced cell death (77). It has also been shown that exposure of Tregs to HIV-gp120 promoted their survival via a cAMP dependent pathway (78), inhibited Treg apoptosis via up-regulation of the anti-apoptotic protein Bcl-2 (79), as well as accumulation of Tregs in peripheral and lymphoid tissues (80). Furthermore, there is an increase in Ki67 (a cell cycle marker) expression in circulating Tregs from untreated, chronically infected patients prior to undergoing ART (81, 82). Third, there may be increased conversion of peripheral naïve CD4 T cells into induced Treg phenotypes. Plasmacytoid dendritic cells (pDCs) represent a small proportion of dendritic cells (DCs) (0.2–0.8% of PBMCs) (83) that have been identified as the main subset of DCs that have the ability to convert allogeneic non-Tregs into CD25+ FoxP3+ Tregs, when exposed to HIV (84). Several studies have shown positive correlations between pDCs and Treg percentages post-therapy (83, 85, Phetsouphanh et al. unpublished data), and indicates that pDCs may play a role in the genesis of peripheral Tregs. Possible reasons for this include (a) development of semi-mature mDCs through HIV interaction that leads to stimulation and proliferation of Tregs (86), which also occurs in SIV infection (87); (b) HIV-stimulated pDCs could induce Treg proliferation by producing indoleamine-2,3-dioxygenase (IDO), and Tregs induced by pDCs have been shown to inhibit maturation of bystander conventional mDCs (84, 88).

## Role of CD39 and Disease Progression during HIV-1 Infection

Two ectoenzymes: CD39 [ecto-nucleoside triphosphate diphosphohydrolase (E-NTPDase)] and CD73 [5′-nucleotidase (5′-NT)] involved in catabolism of extracellular adenosine triphosphate (ATP) have recently been shown to be highly expressed on Tregs in mice, whereas, in humans only CD39 is present and is highly enriched in antigen-specific Tregs (89–91). High levels of extracellular ATP indicate tissue destruction and inflammation. The presence of extracellular ATP can be sensed by purinergic receptors. CD39 can hydrolyze ATP or adenosine diphosphate (ADP) to adenosine monophosphate (AMP) and CD73 can further catabolize AMP to adenosine. Removal of extracellular ATP by CD39 may allow Tregs to migrate to inflamed sites and permit Treg cells to quench ATP-driven pro-inflammatory processes in multiple cell types, in particular, ATP-driven DC maturation. The immunomodulatory effects of ATP removal by CD39 is further enhanced by the generation of adenosine, which binds to A2A adenosine receptor (A2AR) and elicits inhibitory functions of DCs as well as activated T cells (62, 92). This mechanism is widely believed to be important in the observed immunological tolerance of tumors (93).

A consistent feature of Tregs in HIV infections is that they express high levels of CD39, and this high level remains unaltered even with therapeutic interventions (62, 82). Elevated CD39+ Treg frequencies positively correlate with plasma viral load and negatively with CD4 recovery (94, 95). Nikolova et al. demonstrated that a genetic variant of the CD39 gene *ENTPD1* (ecto-nucleoside triphosphate diphosphohydrolase 1) was associated with lower expressions of the CD39 protein, and this led to a slower progression to AIDS (95). High frequencies of CD39+ HIV-specific Tregs were identified in HIV-infected individuals pre-treatment, and low frequencies of CD39− HIV-specific non-Tregs were associated with higher viral load (91). Additionally, blocking of CD39 via monoclonal antibodies eliminated Treg-mediated suppression of CD8+ cytokine production when stimulated with Gag (95). Taking together, CD39+ Tregs may be critical for the inhibition of T-cell associated immune responses, and may control HIV-induced T cell activation, which may reduce HIV replication (91, 96).

Overall, then HIV-1 infection is associated in general with a modest increase in Tregs relative to the conventional CD4 T cells that they normally regulate, and, if anything, may be more active than normal.

## T Follicular Helper Cells and Mechanisms of Action

T follicular helper cells provide help to B-cells in GCs of secondary lymphoid organs and are crucial for GC formation, immunoglobulin class-switch recombination, somatic hyper-mutation, and differentiation of B-cells into long-lived memory B-cells and plasma cells (97). Tfh cells are central to the generation of efficient neutralizing and non-neutralizing antibody responses in HIV infection and will be essential in generating an effective vaccine (98, 99).

T follicular helper cells express high levels of surface markers program death-1 (PD-1) and chemokine CXC receptor 5 (CXCR5), which make them phenotypically distinct from other T helper cell lineages and from peripheral CXCR5+ cells with helper activity for B-cells *in vitro* (as discussed below) (97, 100). However, Tfh cells’ identity as a separate lineage of T helper cells was established when B-cell lymphoma 6 (Bcl-6) was discovered to be necessary and sufficient to drive their differentiation (101, 102).

Naïve CD4 T cells’ multi-step differentiation toward Tfh cells begins with antigen-presenting DCs in the T cell zone (103), stimulating Tfh through TCR, and costimulatory CD28 and ICOS (104). Secretion of IL-6 by DCs serves as a primary signal for the induction of Bcl-6 expression in CD4 T cells in a STAT3-dependent manner, which subsequently drives the expression of Tfh cell signature genes critical for T cell: B-cell interaction, including *Cxcr5*, *Icos*, *Pdcd1*, *Sh2d1a*, and *Cd40l* (105). Another DC secreted cytokine IL-27 induces the expression of transcription factor c-Maf, which cooperates with Bcl6 to enhance the expression of the above Tfh associated genes and induces IL-21 production (106). IL-21 acts to promote Tfh cell differentiation and maintain Tfh cells, probably directly, as well as via its role in inducing Bcl-6 expression and differentiation of GC B-cells, which in turn reinforce Tfh differentiation (107–112). During this process, IL-21 can also induce the expression of B lymphocyte-induced maturation protein 1 (Blimp-1), which is required for the switch from GC B-cells to plasma cells, and activation-induced cytidine deaminase (AID), which is required for class switched recombination (CSR) (112, 113).

T follicular helper differentiation and activity may be regulated at several levels. OX40 (CD134) signaling promotes expression of transcription IRF4 that may cooperate with Bcl6 to maintain Tfh cells (114, 115). High levels of PD-1 on Tfh cells binding to PD-L1 on B-cells provides inhibitory signal to Tfh (116–118). IL-2 signaling prevents Tfh cell differentiation by activating STAT5, which subsequently induces Blimp-1, which represses Bcl6 (119, 120), whereas signaling by interferons or IL-12 may induce T-bet, which complexes with Bcl6 to preemptively repress Blimp-1 (105, 121). Tfh cell differentiation is also reportedly suppressed by CD8+ regulatory cells (122), plasma cells (123), but positively regulated by available antigen presentation (103, 124).

## Follicular Tregs

Follicular Tregs (Tfr) cells were first described as a subset of Tregs that derive from Foxp3+ thymic Tregs and directly repress Tfh cell proliferation and numbers in the GC (111, 125, 126). Tfr and Tfh cells share differentiation and regulation mechanisms, including up-regulation of Bcl6, which instructs the expression of CXCR5, PD-1, and ICOS, and requires CD28 and SAP signaling, as in Tfh cells (111, 125). PD-1/PD-L1 signaling negatively regulates Tfr cells, not only their expansion but also their suppressive ability (127), although the actual number of Tfr in lymph nodes is very small relative to Tfh, in either non-human primate or human lymph nodes (Xu et al. unpublished data). Circulating Tfr, CXCR5+ICOS+Foxp3+ CD4 T cells, have also been described (127). However, whether these cells in peripheral blood have truly come out of a GC reaction and whether they will migrate back to the GC upon recall stimulation needs to be further investigated, to classify them as a distinct Treg subset.

## Circulating Tfh-Like Cells

A subset of circulating memory CD4 T cells bearing the phenotype of CXCR5+, and more stringently CCR7lo, PD-1+, and ICOS+, have been termed “circulating Tfh,” “blood Tfh,” “peripheral Tfh,” or “memory Tfh” cells and are now being intensively studied (128). This reflects the need for surrogate biomarkers in the periphery to correlate with the number of *bona fide* Tfh cells in lymphoid tissue (129, 130). Whether circulating CXCR5+ Tfh-like cells truly represent the memory form of Tfh cells is controversial, although most current evidence suggests that is the case.

First, CXCR5 and PD-1 are stably expressed on these cells rather than a transient response to activation (131). Second, at least a subpopulation of blood CXCR5+ CD4 T cells are highly functional in helping B-cells to survive, to differentiate into plasmablasts, and to produce class switched antibodies upon stimulation *in vitro* or in response to vaccination *in vivo* and this B-cell help is mediated by up-regulation of CD40L or ICOS, and secretion of large amount of IL-21 (130–132). Third, it has been demonstrated in mice that blood CXCR5+ CD4 T cell differentiation is dependent on Bcl6 and ICOS, but not SAP, suggesting that circulating CXCR5+ CD4 T cells are precursors of GC Tfh cells (128). Finally, it has been demonstrated in mice that Tfh cells could revert to memory cells in the absence of antigen and could differentiate into conventional effector cells or Tfh cells upon recall (133, 134). However, blood c-Tfh-like cells and Tfh cells in lymphoid tissue are clearly phenotypically different, particularly with respect to expression of PD-1 and Bcl6 *ex vivo* (135, 136). Recent RNA sequence data also showed that a subset of blood CXCR5+, with the highest helper activity for B-cells *in vitro*, exhibited a gene expression profile more closely related to non-Tfh CD4 T cells than Tfh cells in tonsil (100). Further investigation is required to harmonize the observations and understand the relationship between Tfh cells in lymphoid tissue and different subsets of blood CXCR5+ CD4 T cells.

## Tfh in HIV Infection

In recent years, Tfh cells have been studied intensively in the context of acquired immunodeficiency such as SIV/HIV infection. Early in the 1990s, HIV-1/SIV RNAs had been detected by *in situ* hybridization at high concentrations in the lymph node GCs (20, 21, 137–139). Follicular dendritic cells (FDCs) have been recognized as a major reservoir for virus in lymphoid tissues, facilitating infection of CD4 T cells (140, 141). However, direct evidence for Tfh cells harboring HIV/SIV DNA was only available in the last 2 years (135, 142, 143). Small numbers of Tfh cells were found to be productively infected (135) and replication competent virus could be isolated from infected Tfh cells (143), indicating that Tfh cells are not only a major target of HIV/SIV infection but also a significant CD4 compartment for viral replication and production. This was paradoxical as Tfh cells express very low levels of CCR5 and other HIV/SIV entry coreceptors (135), but they were infected with HIV/SIV at higher or comparable levels, even at a very early stage of infection (135, 143).

More surprisingly, despite being infected with the virus, both cell number and relative percentage of Tfh cells increased during the chronic phase of HIV or SIV infection (135, 142–145). The frequency of Tfh cells correlated with plasma viral load, which suggests that Tfh may be a source of circulating virus (145). The expansion of Tfh cells also correlates positively with the frequency of GC B-cells and antibody production (143, 145). Aberrant Tfh cell expansion is associated with B-cell abnormalities such as polyclonal B-cell activation (146), hypergammaglobulinemia (142, 147), and B-cell driven lymphadenopathy (99, 148, 149). In contrast, broadly neutralizing antibodies (bNAbs) specific for HIV occur very rarely in natural infection (150). bNAbs exhibit unusually high levels of affinity maturation, a result of somatic hyper-mutation (151). Although this was thought to be a by-product of persistent infection, an optimal GC reaction may be required for B-cells to undergo multiple rounds of mutation and selection.

The underlying mechanisms that lead to abnormal GC B-cell responses and antibody production caused by Tfh cell expansion are not fully understood, but at least one mechanism has been proposed. During HIV infection, PD-L1 levels on GC B-cells, but not in memory B-cells, were elevated. Increased PD-1/PD-L1 signaling between Tfh and GC B-cells results in reduction of ICOS expression, which in turn affects downstream IL-21 secretion (118). Since IL-21 is required for GC B-cells survival and differentiation into long-lived plasma cells, GC B-cells receiving inadequate help from Tfh cells may fail to function optimally.

cTfh-like cells, irrespective of how they were defined, were reported to decrease in treatment naïve HIV+ patients (100, 152). This might be a result of CXCR5 internalization in response to elevated serum CXCL13 levels in untreated patients (100) and ART was able to normalize the frequency of cTfh-like cells (100). Such observations are in contrast to the majority of scenarios in primary immunodeficiencies and autoimmune diseases, where blood CXCR5+ CD4 T cell frequencies decrease or increase along with Tfh cells in lymphoid tissue (129, 153). This observation indicates that, if the circulating CXCR5+ CD4 T cells are indeed the memory form of Tfh cells, or traffic out of lymphoid tissue in autoimmune conditions, HIV infection may alter the pattern of Tfh cell trafficking.

The ability of cTfh-like cells’ to help B-cells *in vitro* is compromised, in at least a proportion of HIV+ patients (100, 152). In one report, PD-1+CXCR3−CXCR5+ CD4 T cells in peripheral blood positively correlated with bNAb development in HIV+ donors (131), whereas in another report no such correlation was found (100). This discrepancy likely arises from differences in patient samples and cell subsets studied.

## Treg and Tfh in Tissues

An accumulation of Tregs in gut mucosa and lymphoid tissues has been reported in HIV infection (64, 154). Tregs express the lymph node homing marker CD62L (155, 156) and gut homing integrins α4β7 (157), although the proportion of α4β7+ cells is relatively low, typically around 10% of Tregs (Zaunders et al. unpublished data). The expression of these receptors increases in Tregs following HIV-1 exposure *in vitro* (80). This may explain the accumulation of Tregs in lymphoid and mucosal tissues, where Treg frequencies are much higher than peripheral blood (62, Xu et al. unpublished data). Characteristic molecules such as FoxP3, cytotoxic T lymphocyte antigen 4 (CTLA-4), glucocorticoid-induced-TNFR family related receptor (GITR), and CD25, have been shown to be overexpressed on Tregs in tonsil and lamina propria of duodenal mucosa of untreated patients compared to treated (64, 154).

Other functional Treg markers such as IDO, TGF-β, and CD80 were also markedly increased in tonsillar tissue of untreated patients (154). Furthermore, the prevalence of Treg correlated better with viral load in tissues compared to plasma viremia (158). GALT also represents a major site of HIV replication and CD4 T cell depletion (96, 159, 160). HIV infection leads to a loss in Th17 cells that are vital for mucosal immunity against other pathogens, which may play a role in the increased microbial translocation across the gastrointestinal mucosa leading to systemic immune activation (161, 162). A relative increase in Tregs may play a role in aggravating this effect by inhibiting HIV-specific immune responses in the GALT (163, 164).

There is evidence that Tregs can enter GCs *in vivo*, and suppress CD4 T cell help for B-cells *in vitro* (165) and also directly suppress B-cells (166). The reported mechanism required cell contact, consistent with up-regulation of CXCR5 on Tregs activated *in vitro* and chemotaxis directed by CXCL13. Furthermore, in one study, it was shown that Treg suppression of GC reactions *in vivo* could be counteracted by treatment of mice with antibodies to GITR, TGF-β, or anti-IL-10 (167). As detailed above, there has now been described a small subset of follicular CD4 T regulatory cells, Tfr, which express both Bcl-6 and Foxp3 and exhibit suppressive activity (111, 125, 126). However, these cells appear to be generated during the course of a GC reaction (134), and also may enter the circulation as long-lived memory cells (127, 134). Therefore, these cells may represent a potent feedback mechanism, but it is unclear whether they would normally regulate the conditions during HIV or SIV infection that drive lymph node hyperplasia. Generalized T cell activation during HIV or SIV infection occurs in the T cell areas and regulation of the initial CD4 activation, prior to expression of Bcl-6 and CXCR5, is more likely to be mediated by canonical Tregs.

It has been reported separately that Tregs and Tfh are both increased in lymph nodes in HIV or SIV infection, with the latter possibly showing greater increases, as detailed above, but direct quantitation of both subsets within the same tissue during HIV infection has not been documented. Xu et al. recently studied T cells from lymphoid tissue using fine needle aspiration (FNA) (135) in pigtail macaques. This technique has now been applied to lymph nodes in HIV-infected and uninfected human subjects and it was confirmed that the ratio of Treg to Tfh was <2:1 in lymph node cells from HIV-infected subjects, but was 30:1 in uninfected subjects (Xu et al. unpublished data).

**Figure 1:**
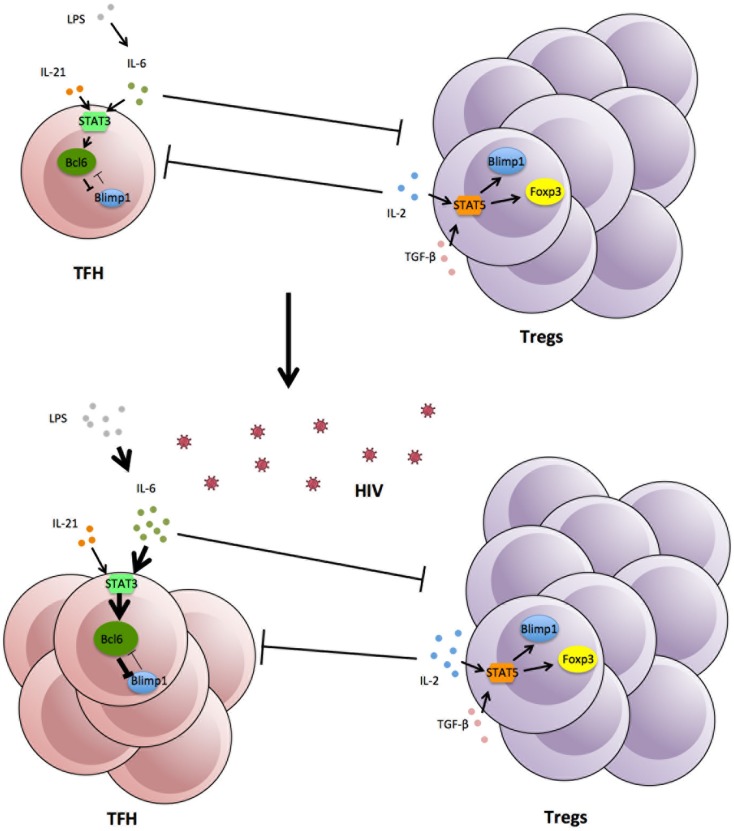
**Expansion of Tfh in lymphoid tissue following HIV-1 infection, associated with increases in cytokines and viral and bacterial products**.

An important consideration is how the increase in Tfh is maintained over such long periods of time, probably years. The lifespan of individual Tfh cells is unknown, although, where studied, they exhibit low levels of Ki67 and are generally not prone to spontaneous apoptosis (142, Xu and Zaunders, unpublished data). Similarly the lifespan of individual GCs is not clear, since they begin to regress by day 14 after primary vaccination in a mouse model and do not greatly increase again with secondary challenge (168), although some studies have reported that GCs can be long lasting in mice, possibly up to 180 days (169, 170). One possibility for long-term elevation of Tfh cell numbers in HIV-1 infection could be due to their own success, if they help generate antibodies that put pressure on the virus, which causes mutations in gp120, which in turn generates neo-antigens, and which in turn generate further immune responses. A striking feature of HIV-1 infection is the continual generation of envelope variants within each patient (171), and later it was found that neutralizing antibody responses were associated with sequential escape mutations (172). Therefore, much more work is required to understand the direct interactions between Tfh and Tregs, how GCs normally regress at the end of an immune response and why this does not happen in HIV-1 infection. Also, the lack of a strictly parallel increase of Tregs and Tfh cells in lymph nodes may indicate that other factors such as cytokines or transcription factors can impact separately on the dynamics of Treg and Tfh in HIV infection (Figure 1).

## Role of Cytokines

IL-6 is a pleiotropic cytokine produced by myeloid cells (monocytes, macrophages, and DCs) (173, 174). It binds to a receptor complex consisting of soluble/transmembrane IL-6 receptor (IL-6R) and the signal-transducing receptor subunit gp130, binding of the receptor potently activates signal transducers and activators of transcription 3 (STAT3), and to a minor extent STAT1 (175, 176). Plasma IL-6 was found to be elevated in HIV infected patients (177) and SIV-infected macaques, but not in SIV-infected African green monkeys, the natural host of SIV (142, 178). ART reduced plasma IL-6 levels, but this reduction never reached levels seen in uninfected donors (179). IL-6 levels in lymph nodes, in contrast, seem to be high in both uninfected and HIV infected samples (174, 179), although it was reported that IL-6 mRNA levels were increased in lymph nodes from macaques as early as 7 days post SIV infection (180). However, HIV itself does not seem to be the direct driver of IL-6 production (179, 181, 182). Instead, LPS alone markedly induced IL-6 production at low concentrations (181, 182). Increased plasma LPS is not only a property of pathogenic SIV infection but has also been reported in progressive HIV infection (159). Again, ART reduced plasma LPS level significantly but failed to reach levels found in uninfected donors (159). Therefore, persistently high levels of LPS despite ART may result in persistently high levels of IL-6, and subsequently Tfh cell accumulation in chronic HIV/SIV infection (183).

Transforming growth factor-β is a binary cytokine in CD4 T cell induction. Together with IL-2, it stimulates the differentiation of peripheral Tregs via STAT5; with IL-6, it inhibits the generation of peripheral Tregs but induces the development of Th17 cells via STAT3 (184, 185). The reciprocal relationship between Th17 cells and Tregs has been well documented (184, 186). However, the relationship between Tfh cells and Tregs remains elusive. Oestrich et al. showed that in IL-2 limiting conditions Th1 cells can upregulate BCL-6, which converts these cells into Tfh-like cells with similar gene profile including up-regulation of CXCR5 (121). High levels of exogenous IL-2 have been reported in HIV infected subjects with high viral load (187, 188). As Tregs are known to mop up IL-2 for homeostatic proliferation, this may explain both the accumulation of Tregs and expansion of Tfh in tissue. Tsuji et al. reported on the generation of Tfh cells from Foxp3+ Tregs in gut Peyer’s patches, but not in spleen or lymph nodes (189). However, more work is required to confirm this finding.

Regulatory T cells and Tfh share an extremely important property, namely, low expression of the IL-7 receptor alpha chain, CD127 (51, 135), which distinguishes them homeostatically from the vast majority of CD4 T cells. This feature may be highly relevant to their ascendency during chronic HIV-1 infection if damage to lymph nodes (40) affects IL-7 signaling.

## Role of Transcription Factors

Bcl-6 and BLIMP-1 are key antagonistic transcriptional regulators of effector and memory differentiation in CD8+ and CD4 T cells, but were first identified as critical regulators of B-cell maturation and memory formation, determining cell fate decisions (101, 190). Bcl-6 and BLIMP-1 have been studied in HIV infection, and BLIMP-1 is highly expressed at both the mRNA and protein levels in CD4 T cells in patients with chronic HIV infection compared to LTNP (191). The lower expression of BLIMP-1 in CD4 T cells from LTNP correlates with lower levels of exhaustion in CD4 T cells found in LTNP (191). The expression of BLIMP-1 can be modulated at the translational level by microRNA-mir9 and Seddiki et al. demonstrated that BLIMP-1 levels decreased following treatment with pre-mir-9, while IL-2 expression was increased. Levels of mir-9 were also found to be elevated in LTNP compared to chronically infected subjects (191, 192). BLIMP-1 has also been found to be required for effector Treg differentiation and is essential for IL-10 production (193). Therefore, the level of BLIMP-1 expression in Tregs in chronically infected subjects and LTNP should be investigated to further delineate the importance of this transcription factor in HIV infection. Also, the antagonistic effects of Bcl-6 and BLIMP-1 may present a therapeutic target for the manipulation of T helper subset fate decision.

## Tregs/Tfh as Potential Targets of HIV Immunotherapy

As Tregs and Tfh cells play crucial roles in homeostatic immune responses and the dysregulation of these cells due to HIV-infection causes severe morbidity, therefore Treg and Tfh cells are of interest as potential targets for immunotherapeutic intervention. Many strategies have been implemented to influence the frequency and function of these cells, such as inhibition of specific enzymes, monoclonal antibody (mAb) therapy, and cytokine based clinical trials, as detailed below.

The enzymatic activity of IDO has the ability to influence the Th17/Treg balance, and can enhance the suppressive activity of Tregs. Thus, modulation of IDO in disease is of therapeutic interest. In an animal model of HIV-1 encephalitis, inhibition of IDO via 1-methyl-d-tryptophanh (1-MT) enhances the generation of HIV-specific cytotoxic T cells, which led to the destruction of macrophages in the brain (194). In other observations, IDO seems to synergize with therapy to control viral replication in lymph nodes and plasma of macaques infected with SIV (195). The inhibition of both IDO and CTLA4 in combination has been shown to transiently reduce the kynurenine/tryptophan ratio, increase Th1 proliferation and block Treg suppressive functions. A side effect of this combination therapy, however, resulted in fulminant diabetes with severe infiltration of lymphocytes in the pancreas (196). Taking these previous findings into consideration, potential IDO inhibitors need to be studied intensively in the context of HIV therapy.

Program death-1 is an important marker that modulates the inhibitory pathway, which regulates the T-cell receptor signaling (197). This has been studied intensively in chronic viral infections (198–201). PD-1 is expressed at high levels on HIV-specific T cells during HIV infection, and correlates with plasma viral load, reduced cytokine production, and impedes proliferation of HIV-specific CD8+ T cells (202). PD-1 blockade enhanced the capacity of HIV-specific CD8+ T cells to survive and proliferate during infection, as well as intensifying HIV-specific CD8+ T cells responses (202). PD-1/PD-ligand axis enables the conversion of Th1 cells into Tregs, thus by blocking PD-1 with a mAb may aid the initial response to HIV in early infection (203). Consistent with a role for PD-1/PD-L1 and PD-L2 in Tfh function (116), it has been shown that PD-1 blockage on PD-1 high Tfh cells co-cultured with B-cells significantly inhibits IgG production (204). As Tfh cells accumulate in HIV infection and these cells predispose to B-cell related morbidities, PD-1 blockade could be considered as potential therapeutic intervention.

Cytotoxic T lymphocyte antigen 4 (CD152) is another target for therapeutic intervention. The administration of anti-CTLA-4 blocking antibodies was not detrimental and had beneficial virological effects in SIV-infected ART treated macaques. Decreases in IDO, TGF-β, and viral RNA expression in tissues were observed (205). However, in untreated SIV infection, CTLA-4 inhibition did not restore SIV-specific immune responses and there was an increase in viral replication and CD4 depletion, particularly at mucosal sites (206). It was found that even at the earliest stages of primary HIV-1 infection, Gag-specific CD4 T cells were dominated by expression of CLTA-4 (18), and it was found that *in vitro* blockade of CTLA-4 significantly increased CD4 T cell proliferation and improved cytokine secretion from HIV-specific CD4 T cells responding to cognate antigen (207). It has also been shown that combination blockade of PD-1 and CTLA-4 reduced Treg activity in cancer (208). However, whether the same approach in HIV infection would yield similar results, remains to be ascertained.

Cytokine based clinical trials have been implemented in the past to facilitate the restoration of T cells in HIV infection. IL-2 is a critical cytokine needed as a strong stimulatory signal for Treg development and function (209, 210). IL-2 was the first candidate cytokine used as an immunotherapeutic agent to boost total CD4 cell counts, although one of the benchmarks of treatment was an increase of CD4+CD25+ T cells, which potentially included Treg cells. Two major phase III clinical trials were conducted, but despite substantial increases in CD4 T cell count, IL-2 in addition to ART yielded no clinical benefit compared to ART alone, in either study (211). These trials showed predominant increases in CD4+CD25+CD127lowFoxP3+ cells, and these cells exhibited molecular and suppressive functions such as those found in Tregs (75). However, there was also a lack of protective effect of IL-2 expanded CD4 T cells on HIV disease progression. In addition, there were potential deleterious effects observed in treated patients relating to cardiovascular and inflammatory events (212). A possible explanation for this is the expansion of suppressive Tregs with truncated STAT5 expression, rendering these IL-2 expanded cells ineffective in protecting against disease progression (96, 212). Thus, other trials using other immunological-based compounds must carefully monitor the phenotype and function of the expanded CD4 T cells.

IL-7 immunotherapy was also developed for HIV infection, first conducted in animal models, where increases in CD4 T cell counts were observed in the absence of immune activation (213, 214). Contrary to IL-2 based immunotherapy, administration of IL-7 resulted in the expansion of CD4 T cells without increasing the frequency of immune-suppressive Tregs, consistent with the low levels of the IL-7 receptor (CD127) expressed on Tregs (51). Also, in one study, *in vitro* incubation in the presence of IL-7 reduced the suppressive activity of Tregs isolated from HIV+ subjects (69), suggesting that IL-7 therapy may have another effect to further boost conventional T cell responses. Due to these differences in responsiveness to IL-7, immunomodulation using various strategies involving either blocking of the receptor to suppress responses or addition of IL-7 to boost responses is currently being investigated in a number of other clinical situations, including autoimmunity, cancer vaccines, and transplant tolerance [reviewed in Ref. (215)].

IL-21 is a pleiotropic cytokine that is important for T cell and B-cell proliferation and maintenance (216) and is produced most abundantly by Th17, Tfh, and natural killer T (NKT) cells. As discussed above, Tfh cells require this cytokine to enhance proliferation and function. Previous animal models have also shown that IL-21 had stimulatory effects on NK cells and CD8+ T cells, and this effect leads to anti-tumor activity (217). Now, IL-21 has been used in phase I and II trials in cancer and early results demonstrated that recombinant IL-21 administration has an acceptable safety profile and has demonstrated encouraging activity in early phase renal cell carcinoma and melanoma trials (218). This makes IL-21 a potential agent for Treg/Tfh modulation, as IL-21 has inhibitory effects on Treg differentiation via the reduction of IL-2 production from other CD4 T cells (219). Since Tfh cells require IL-21 for homeostatic proliferation and are suited to function in low IL-2 conditions, strategies to modulate IL-21 signaling could be used to modulate Treg/Tfh dynamics in HIV infection.

## Conclusion

HIV-1 infection leads to chronic activation of T cells, B-cells, and myeloid lineage cells within lymphoid tissue, as a result of the combined effects of the host immune response, the increased presence of viral and bacterial products that drive inflammation, and homeostatic processes that fail to bring inflammation under control. There are increases in the number of both Tregs and Tfh, but in the face of continuing viral replication, the feedback regulation by Tregs does not prevent the florid hyperplasia associated with increased numbers of Tfh and GC B-cells. ART may ameliorate the lymphocyte activation mostly, but not completely. Therapeutic strategies aimed at limiting Tfh activity, or modulating Tregs, should be investigated for potential benefits to boost CD4 reconstitution without unduly boosting Tfh and B-cell hyper-reactivity, or Treg activity.

However, the aim of therapeutic interventions will require very careful consideration due to the complexity of the roles of Tfh and Tregs in pathogenesis. In the case of Tfh, generation of neutralizing antibodies through directed Tfh and B-cell vaccination is a highly desirable outcome (98, 99), but this must be balanced by avoiding excessively increased activation of CD4 T cells and additional GCs as reservoirs of HIV. Similarly, increased Treg activity under HAART may be advantageous in reducing atherosclerosis (220) given the known increased risk of cardiovascular disease in HIV patients, associated with increased inflammation (221), but must be balanced against a need for improved immune reconstitution. Only very detailed studies of these processes will allow rational development of optimal therapy.

## Conflict of Interest Statement

The authors declare that the research was conducted in the absence of any commercial or financial relationships that could be construed as a potential conflict of interest.
